# The association between periodontal disease and lung cancer: a re-evaluation of independent causal relationships in the presence of smoking as a confounder and an exploration of potential mechanisms

**DOI:** 10.3389/fcimb.2026.1964724

**Published:** 2026-09-22

**Authors:** Wenwen Liu, Yanli Yang, Dongmei Zhang

**Affiliations:** Respiratory Department, First Hospital of Jilin University, Changchun, China

**Keywords:** biological mechanisms, dose-response relationship, lung cancer, oral-lung axis, periodontal disease, smoking confounding

## Abstract

Periodontal disease and lung cancer are major global health burdens, and epidemiological evidence suggests a potential association between them. However, whether this relationship is independent of smoking—a strong common risk factor—remains controversial. This review systematically summarizes current evidence on the association, with emphasis on smoking confounding and underlying biological mechanisms. Meta-analyses consistently show a significant positive association, with adjusted hazard ratios ranging from 1.24 to 1.71 and dose-response relationships across disease severity and tooth loss. After rigorous statistical adjustment for smoking, most large cohort studies still show a robust association, though findings in never-smokers are inconsistent, suggesting possible residual confounding. Mechanistically, the “oral-lung axis” links periodontitis to lung carcinogenesis via three pathways: microbial translocation of pathogens such as Fusobacterium nucleatum, chronic inflammation-driven immune dysregulation involving Treg and IL-6, and epigenetic modifications including shared DNA methylation sites. Smoking may also act synergistically with periodontitis to amplify carcinogenic risk. Future research should prioritize precisely quantified smoking exposure, formal interaction analyses, and longitudinal multi-omics studies to support causal inference. This review provides a theoretical foundation for incorporating oral health assessment into lung cancer prevention strategies.

## 1 Introduction

Periodontal disease (PD) is traditionally defined as a chronic inflammatory disease affecting the tissues surrounding and supporting the teeth (Nocini et al., 2020; Agarwal et al., 2025). It is an oral infection and inflammatory disease caused by pathogenic microorganisms in dental plaque on the tooth surface; these pathogens trigger a host-mediated immune response, which leads to progressive destruction of soft tissues and alveolar bone (Luchian et al., 2021; Grundner et al., 2022). As one of the most common oral diseases worldwide, periodontal disease is the second-largest oral health burden after dental caries (Rocha et al., 2021; Leija-Montoya et al., 2023). Its prevalence continues to rise globally, raising serious public health concerns (Papapanou and Susin, 2017). According to epidemiological studies, periodontal disease affects approximately 20% to 50% of the global population (Nazir, 2017; Amato et al., 2022). In developed countries, its prevalence ranges from about 30% to 50%, but only about 10% of patients present with severe forms of the disease (Han et al., 2016). Lung cancer is one of the most common malignant tumors in most countries worldwide and is also the leading cause of cancer-related deaths (Mao et al., 2016; Smolarz et al., 2025). In the United States, lung cancer has long been the leading cause of cancer-related deaths among both men and women, and it is one of the deadliest cancers globally (Barta et al., 2019; Casazza et al., 2026). In terms of epidemiological trends, lung cancer has evolved from a relatively rare disease into a global public health crisis (Mao et al., 2016). It affects as many as 1 in 15 men and 1 in 17 women (Athanasiou et al., 2025). The geographic and temporal patterns of lung cancer incidence and mortality are primarily determined by tobacco consumption patterns (Malhotra et al., 2016).

In recent years, many epidemiological studies have begun to focus on the potential association between periodontal disease and the risk of lung cancer. Several meta-analyses have shown that patients with periodontal disease have a significantly increased risk of developing lung cancer, with adjusted hazard ratios ranging from 1.24 to 1.71, and a clear dose-response relationship has been observed (Zeng et al., 2016; Michaud et al., 2017; Kesharani et al., 2022; Zhang et al., 2023). However, establishing a clear causal relationship remains a major challenge (Higham and Scannapieco, 2024). The core difficulty lies in the fact that smoking is a strong common risk factor for both conditions—smoking increases the risk of developing and progressing periodontitis by 85%, and its population-attributable fraction for lung cancer is as high as 63.73% (Leite et al., 2018; Wang et al., 2022). This makes it considerably challenging to isolate the independent effect of periodontal disease. Some researchers have even suggested that the observed association may merely represent a “proxy” effect of smoking (Zhang et al., 2023). Some studies have observed a significant association between severe periodontitis and lung cancer risk even after strictly adjusting for confounding factors such as smoking and socioeconomic status, while others have found no significant association among never-smokers (Michaud et al., 2018; Zhang et al., 2023). This review aims to systematically summarize the current epidemiological evidence and conduct an in-depth analysis of the impact of smoking—a key confounding factor—on the association between periodontal disease and lung cancer, as well as its underlying mechanisms. We hope that this synthesis and re-evaluation of the evidence will provide a theoretical foundation for future studies on causal inference, the exploration of biological mechanisms, and clinical translation, and will lay the groundwork for incorporating periodontal health into lung cancer risk management strategies.

### 1.1 Literature search strategy

Given the narrative and mini-review nature of this work, we performed a focused literature search in PubMed, Web of Science, and Scopus through August 2026, using combinations of terms related to periodontal disease (e.g., periodontitis, tooth loss), lung cancer, and smoking. We prioritized large-scale cohort studies, meta-analyses, and key mechanistic investigations published in English. We identified additional relevant articles through hand-searching reference lists. After title/abstract screening and full-text assessment, we excluded articles without original data (e.g., editorials, conference abstracts), animal studies not relevant to human cancer pathways, and epidemiological studies that did not adjust for smoking. This process yielded approximately 60 core references directly supporting our three thematic axes: epidemiological confounding, microbial translocation, and immune/epigenetic mechanisms.

## 2 The epidemiological association between periodontal disease and lung cancer

Meta-analyses consistently demonstrate a positive association between periodontal disease and lung cancer risk. A clear dose-response relationship exists across disease severity and tooth loss. However, studies show significant heterogeneity. The results range from no statistical significance to a strong association. This highlights the continuing uncertainty regarding whether this relationship is independent of the confounding factor of smoking. Therefore, further investigation using more refined analytical methods is warranted to better characterize this association and assess its independence from confounding.

### 2.1 Assessment of the strength of association in cohort studies and meta-analyses

Several large-scale meta-analyses have consistently shown that periodontal disease is significantly positively associated with lung cancer risk; however, the pooled effect size shows some heterogeneity due to differences in study design, population characteristics, and diagnostic criteria among the included studies. A meta-analysis incorporating five cohort studies (321,420 participants) used a fixed-effects model and, after adjusting for confounding factors, showed a significant association between periodontal disease and the risk of new-onset lung cancer, with a hazard ratio of 1.24 (95% CI: 1.13–1.36) (Zeng et al., 2016). Another meta-analysis encompassing 12 studies reported a higher relative risk, suggesting that, after adjusting for age and smoking status, the incidence of lung cancer is approximately twice as high among patients with periodontal disease (Kesharani et al., 2022). A systematic review of the literature from 2010 to 2022 included 7 studies (194,850 participants) and reported a lung cancer risk ratio of 1.41 (95% CI: 1.32–1.52) (Verma et al., 2023). A systematic review incorporating 8 studies showed that the hazard ratio for periodontitis and lung cancer was 1.24 (95% CI: 1.06–1.45), while a meta-analysis based on 26 cohort studies indicated that the relative risk (RR) of self-reported periodontal disease and lung cancer was 1.19 (95% CI: 1.09–1.31) (Corbella et al., 2018; Duan et al., 2026). However, some large health insurance database studies have reported discrepancies. For example, a retrospective cohort study in Taiwan involving 86,886 patients with chronic periodontitis reported an overall RR of only 1.01 (95% CI: 0.90–1.12), which was not statistically significant (Shen et al., 2025). In contrast, the South Korean National Health Insurance Cohort Study reported a higher adjusted hazard ratio (Kim et al., 2024). These differences in effect sizes may stem from variations in population characteristics, follow-up duration, and periodontal disease diagnostic criteria.

### 2.2 Dose-response relationship between clinical phenotypes of periodontal disease and lung cancer risk

Epidemiological evidence suggests that the association between periodontal disease and lung cancer is not a simple “yes or no” relationship but may instead exhibit dose-response characteristics based on clinical severity and specific phenotypes. Regarding the severity of periodontitis, the Health ABC cohort study showed that severe periodontal disease was significantly associated with an increased incidence of lung cancer, with an adjusted HR of 3.12 (95% CI: 1.45–6.72) (da Silveira et al., 2026). Another cohort study of older adults also confirmed that severe periodontal disease is associated with a significantly increased risk of lung cancer (HR = 2.33; 95% CI: 1.51–3.60) (Michaud et al., 2018). A South Korean cohort study further refined the risk stratification, finding that patients with mild and moderate-to-severe chronic periodontitis had a 2.45-fold and 2.10-fold increased risk of lung cancer, respectively (Kim et al., 2024). Pooled data from meta-analyses also support this graded relationship, with the HR for lung cancer rising from 1.58 to 2.39 as the severity of periodontal disease increases (Zhang et al., 2023). Regarding the tooth loss phenotype, a meta-analysis incorporating 12 cohort studies showed that tooth loss is associated with an increased risk of lung cancer (RR = 1.69; 95% CI: 1.46–1.96), and a clear dose-response relationship exists: for every additional 5 missing teeth, the risk of lung cancer increases by 10% (Chen et al., 2020). A nested case-control study in the southeastern United States found that the odds ratio for lung cancer among individuals with more than 10 missing teeth was 1.64 (95% CI: 1.00–2.69) (Yoon et al., 2019). A study using the South Korean National Health Insurance Database also confirmed that tooth loss is significantly associated with both the risk of lung cancer incidence and the risk of death (Lee and Kim, 2025). However, some study results are inconsistent. For example, in the Health ABC study, tooth loss was associated with a reduced incidence of lung cancer (HR: 0.96; 95% CI: 0.93–0.99), suggesting that the predictive value of tooth loss as a marker of cumulative periodontal damage may vary across populations (da Silveira et al., 2026). Furthermore, specific bacterial loads also exhibit a dose-response relationship. The ARIC cohort study showed that for each increase of one quartile in antibody levels against the Orange-related bacterial complex—a group of bridging periodontal pathogens, including Fusobacterium nucleatum, that facilitate the establishment of more virulent late colonizers according to Socransky’s classification (da Silva et al., 2023; Santi-Rocca et al., 2025)—the HR for lung cancer risk was 1.15 (95% CI: 1.02–1.29) (Zhou et al., 2023). The existing epidemiological evidence is statistically robust, but residual confounding factors must still be ruled out before a biologically independent association can be conclusively established.

## 3 In-depth analysis and statistical control of smoking confounding effects

Smoking is the most important modifiable risk factor common to both periodontal disease and lung cancer. Its dual pathogenic role constitutes the greatest source of confounding in studies examining the association between the two. At the biological level, smoking not only directly increases the risk of lung cancer but is also an independent risk factor for the onset and progression of periodontitis. A systematic review showed that smoking increases the risk of periodontitis by 85% (HR = 1.85) (Leite et al., 2018). Smoking disrupts periodontal homeostasis by interfering with the host inflammatory response, altering the subgingival microbial community, and impairing tissue healing capacity, leading to a significantly higher prevalence of moderate-to-severe periodontal attachment loss among smokers (Brennan et al., 2017; Apatzidou, 2022). At the microbiome level, regardless of periodontal health status, subgingival plaque in smokers shows enrichment of pathogenic bacteria and altered diversity, creating a “vulnerable” periodontal microecological environment (Jiang et al., 2020; Tamashiro et al., 2023). Furthermore, daily smoking volume is associated with periodontal clinical parameters and salivary biomarker levels in a dose-dependent manner (Mohanty et al., 2019; AlZamil and AlQutub, 2023). At the sociobehavioral level, smoking coexists with lower oral health literacy and poor healthcare-seeking behaviors; for example, individuals who seek care only when symptoms appear have a poorer oral health-related quality of life (OR = 1.65) (Collins et al., 2019). This pattern may simultaneously delay both periodontal treatment and early lung cancer screening, further obscuring the true relationship between exposure and outcomes. Therefore, when assessing the association between periodontal disease and lung cancer, it is essential to fully consider the multiple confounding mechanisms of smoking across the dimensions of molecular pathology, microbiome remodeling, and health behaviors.

Although smoking is a strong confounding factor, multiple high-quality epidemiological studies have consistently shown that the positive association between periodontal disease and lung cancer remains robust after statistically rigorous adjustment for smoking. A meta-analysis incorporating 12 studies showed that, after adjusting for age and smoking, the incidence of lung cancer among patients with periodontal disease was still approximately twofold higher (Kesharani et al., 2022). A meta-analysis of 5 cohort studies and 321,420 participants yielded an adjusted pooled HR of 1.24 (95% CI: 1.13–1.36) (Zeng et al., 2016). The Korea National Health Insurance Cohort Study (n = 72,658), after adjustment via multivariate Cox regression, showed an HR of 2.27 (95% CI: 1.94–2.65), with the risk increasing 2.45-fold and 2.10-fold in the mild and moderate-to-severe periodontitis groups, respectively (Kim et al., 2024). However, some systematic reviews have pointed out that no significant association between periodontal disease and lung cancer was found among never smokers (HR = 1.00, 95% CI: 0.76–1.31), suggesting that the overall association may be partially driven by smoking as a confounder (Zhang et al., 2023). The available evidence generally supports the notion that periodontal disease is an independent risk factor for lung cancer. However, effect sizes typically diminish after fine-tuning for smoking-related indicators compared with crude estimates, and results from subgroup analyses in non-smokers are inconsistent, possibly due to differences in sample size, periodontal disease diagnostic criteria, and the definition of “non-smokers” (Zeng et al., 2016; Zhang et al., 2023; Kim et al., 2024). Intervention studies targeting nonsmokers are currently underway to assess changes in lung function following improvements in oral hygiene; these studies will provide functional evidence to validate the biological plausibility of the nonsmoking pathway (Kühnisch et al., 2023). This field still requires further confirmation through large-scale, prospective studies with strictly defined smoking status.

Exploring the interaction between periodontal disease and smoking is crucial for understanding the combined carcinogenic mechanism. Existing evidence suggests a possible positive synergistic effect. A meta-analysis showed that the strength of the association between periodontal disease and lung cancer in the subgroup of smokers (OR = 1.47) was significantly higher than the overall estimate (OR = 1.35), implying that smoking may amplify the carcinogenic effect of periodontal disease (Wang et al., 2020). Studies on biological mechanisms support this view: smokers exhibit increased epithelial thickness and reduced collagen density in periodontal tissues, as well as abnormal expression of tight junction proteins in gingival crevicular fluid, which may compromise the barrier function of the oral-lung axis and facilitate the translocation of periodontal pathogens and their toxins to the lungs (Güney et al., 2023; Reddy et al., 2025). Smoking alters the host’s immune response network to periodontal pathogens; in smokers with periodontal disease, the interaction between clinical variables and the bacterial antibody profile is significantly enhanced (Nagarajan et al., 2017). However, some studies have not found significant multiplicative or additive interactions or have observed differing interaction patterns under specific genotypic backgrounds. For example, the association between NOS3 gene polymorphisms and periodontal disease is significant only in nonsmokers and is masked in smokers (Mazurek-Mochol et al., 2018). Currently, epidemiological data on these interactions remain relatively limited; most studies have conducted only stratified analyses without formally testing interaction terms. Future research should employ specialized statistical methods to quantify the combined effects of these factors to determine whether a superadditive carcinogenic risk exists.

## 4 Biological mechanisms linking periodontal disease and lung cancer: from microbial translocation to epigenetic regulation

The association between periodontal disease and lung cancer is supported not only by epidemiological data but also by a gradually emerging biological rationale elucidated through multidimensional research in microbiology, immunology, and epigenetics (**Figure 1**). The biological link between periodontal disease and lung cancer can be explained by the “oral-lung axis” theory, which posits that periodontal pathogens can migrate from the oral cavity to the lungs via microinhalation or the bloodstream, thereby directly contributing to lung cancer development and progression (Baima et al., 2024; Jin et al., 2026). In this process, *Fusobacterium nucleatum* is considered a key candidate carcinogenic bacterium; its abundance is significantly and positively correlated with lung cancer risk, independent of other confounding factors (Zhang et al., 2023). Animal studies have further confirmed that oral infection with Fusobacterium nucleatum leads to a significant decline in lung function in mice, along with markedly elevated levels of inflammatory factors such as IL-1β, IL-6, IFN-γ, and TNF-α, as well as tissue-damaging factors such as matrix metalloproteinase-8 and neutrophil elastase in lung tissue. Furthermore, the bacterium can be detected in lung tissue, suggesting direct pulmonary pathogenicity (Li et al., 2024). Furthermore, the airway microbiome of lung cancer patients is often characterized by an enrichment of *Streptococcus*, *Veillonella*, and the Bacteroides phylum, along with reduced α-diversity in the oral microbiome; this dysbiotic profile may serve as an early warning sign for lung cancer development (Zhou et al., 2023; Liu et al., 2026).

**Figure 1:**
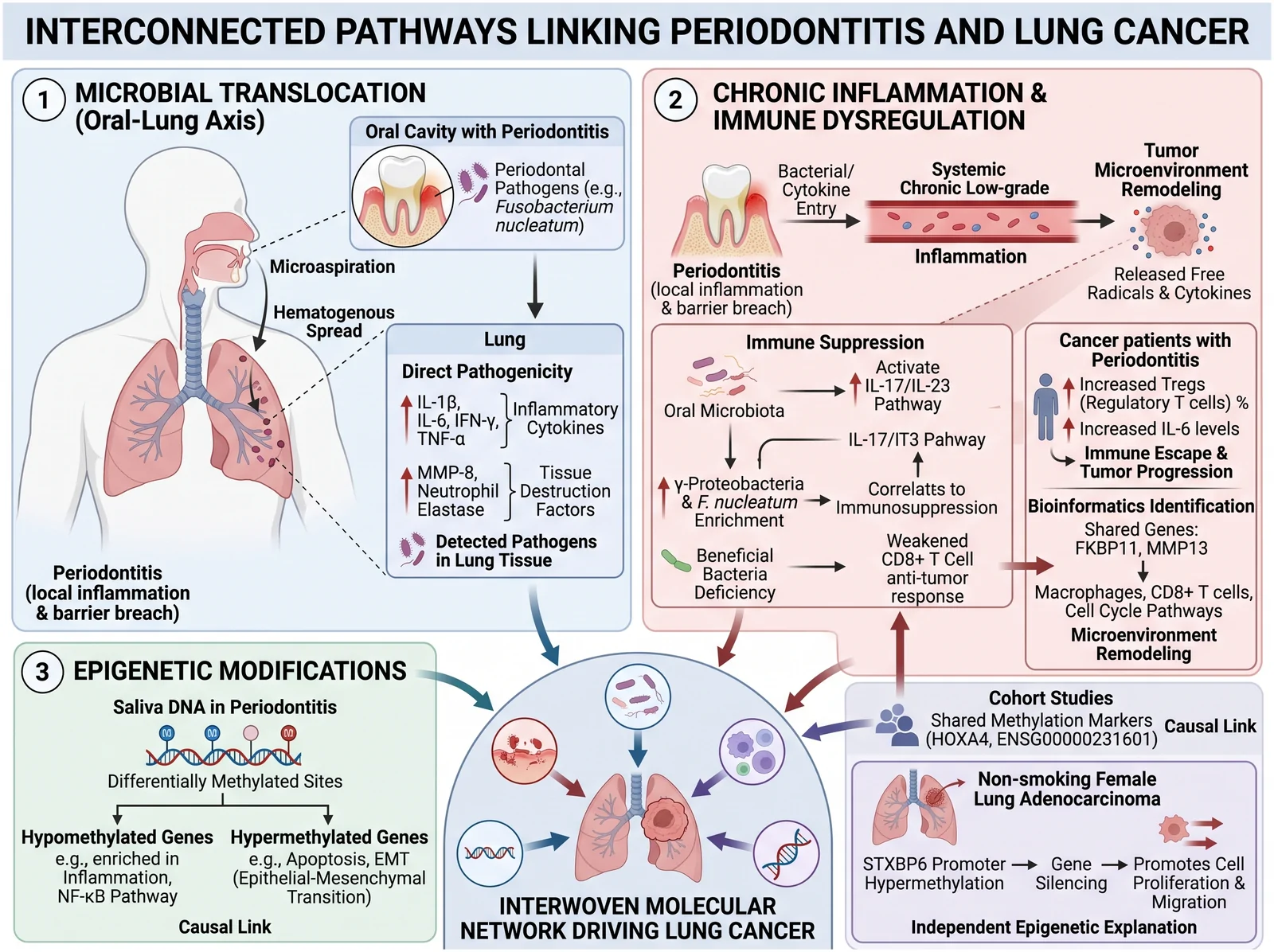
Schematic overview of the biological mechanisms linking periodontal disease to lung cancer. The figure illustrates three interconnected mechanistic pathways through which periodontal disease may promote lung carcinogenesis via the "oral-lung axis." Microbial translocation: Periodontal pathogens, particularly Fusobacterium nucleatum, migrate from the oral cavity to the lungs via microinhalation or hematogenous routes, inducing local inflammation and tissue damage. Chronic inflammation and immune dysregulation: Periodontitis disrupts local barriers, allowing bacteria and inflammatory mediators to enter the circulation, leading to systemic low-grade inflammation, Treg expansion, IL-6 elevation, and tumor microenvironment remodeling. Epigenetic modifications: Periodontal disease-associated DNA methylation alterations at shared sites (e.g., HOXA4, STXBP6) may contribute to lung cancer risk.

Chronic inflammation-mediated systemic immune dysregulation is another core mechanism by which periodontal disease drives lung cancer progression. As a chronic, multifactorial inflammatory disease, periodontitis can disrupt local barriers, allowing bacteria, virulence factors, and inflammatory mediators to enter the systemic circulation and induce a chronic, low-grade systemic inflammatory state (Baima et al., 2024). Through the release of free radicals and cytokines and the degradation of the extracellular matrix, it may contribute to a microenvironment conducive to tumorigenesis and metastasis (Baima et al., 2024). Within the tumor microenvironment, the oral microbiome can activate the IL-17/IL-23 pathway, promote chronic inflammation, and reshape the immune landscape (Liu et al., 2026). In patients with non-small cell lung cancer, the enrichment of γ-proteobacteria and Fusobacterium nucleatum is associated with immunosuppression, while the absence of beneficial bacteria such as *Akkermansia muciniphila* weakens the antitumor response of CD8^+^ T cells (Liu et al., 2026).

Synergistic effects at the immune regulatory level are particularly noteworthy: the proportion of regulatory T cells in the peripheral blood of patients with both cancer and periodontitis is significantly higher than in patients with cancer alone or periodontitis alone, suggesting that periodontitis may enhance the immunosuppressive microenvironment by inducing Treg expansion, thereby promoting tumor immune evasion (Kajihara et al., 2022). At the same time, peripheral blood IL-6 levels were significantly elevated in the cancer group and further increased in the group with both cancer and periodontitis, suggesting that periodontitis may contribute to cancer progression by exacerbating systemic inflammation (Kajihara et al., 2022). Integrated bioinformatics analysis further identified FKBP11 and MMP13 as cross-interacting genes between periodontitis and lung adenocarcinoma (Wang et al., 2024). Mechanistic analysis suggests these genes may contribute to the comorbidity of the two diseases by influencing macrophages, M1 polarization, and CD8^+^ T-cell activity, as well as disrupting immune-related and cell-cycle pathways (Wang et al., 2024). However, this molecular association may not reflect a direct causal relationship but may instead indirectly affect the tumor microenvironment by remodeling immune cell function and dysregulating cell-cycle control (Wang et al., 2024).

Epigenetic modifications, particularly DNA methylation, are regarded as a key bridge between environmental exposures and genomic alterations in lung cancer (Barros et al., 2018; Jurkowska, 2024). Salivary DNA from patients with periodontitis contains many differentially methylated sites; among these, hypomethylated genes are enriched in pathways regulating inflammatory responses and leukocyte activation, and in transcription factors such as NF-κB, while hypermethylated genes are involved in apoptotic signaling and epithelial-mesenchymal transition. These epigenetic alterations may create a favorable environment for cancer development (Petrenya et al., 2026). In lung cancer, the CLUE II cohort study found that methylation levels at 14 CpG sites in the HOXA4 gene region were significantly associated with lung cancer risk, and that a specific CpG site in the ENSG00000231601 gene was also associated with lung cancer risk (Mulvaney et al., 2024). Because these sites are also associated with periodontal disease, they may play a mediating role in the association between periodontal disease and lung cancer (Mulvaney et al., 2024). In non-smoking female patients with lung adenocarcinoma, high methylation of the STXBP6 gene promoter leads to its silencing, thereby promoting cell proliferation and migration, providing an independent epigenetic explanation for the association between periodontal disease and lung cancer in non-smokers (Lenka et al., 2017). Because of their early appearance, high disease specificity, and stability, epigenetic markers have become promising targets for early lung cancer detection (Lianidou, 2021; Jurkowska, 2024).

### 4.1 Direct genotoxicity: microbial acetaldehyde production

Beyond microbial translocation and chronic inflammation, a third mechanistic pathway links periodontal dysbiosis to lung carcinogenesis through the direct genotoxic effects of microbe-derived acetaldehyde (Nikolić et al., 2025). Acetaldehyde, classified as a Group 1 human carcinogen, is a metabolic byproduct of ethanol that oral microorganisms with alcohol dehydrogenase (ADH) enzymes can produce locally (Na and Lee, 2017; O'Grady et al., 2020). Key acetaldehyde-producing taxa within the oral cavity include *Neisseria species*, which exhibit ADH activity more than 100-fold higher than other genera, as well as *Streptococcus mitis*, *Rothia mucilaginosa*, *Prevotella histicola*, and *Candida species* (Muto et al., 2000; Moritani et al., 2015).

Acetaldehyde is an electrophilic molecule that readily reacts with nucleosides to form DNA adducts, leading to point mutations, sister chromatid exchanges, and genomic instability (Mizumoto et al., 2017). This DNA damage can activate oncogenic signaling pathways such as MAPK and NF-κB while inhibiting DNA repair mechanisms (Zhang et al., 2025). This microbial metabolic pathway has systemic implications: acetaldehyde produced in the oral cavity can reach the lungs via microaspiration or the bloodstream, potentially inducing direct DNA damage in pulmonary epithelial cells (Chen et al., 2018). This mechanism can operate independently of the inflammation-driving pathways mentioned above, or it can act synergistically.

Smoking appears to potentiate this pathway. Tobacco smoke can enhance oral bacterial acetaldehyde production from alcohol, and the combination of smoking and alcohol consumption may modify the oral microflora to produce higher salivary acetaldehyde levels (Vishwakarma and Verma, 2021). This interaction suggests that smoking may amplify periodontal disease-associated carcinogenic risk not only through confounding and immune modulation, but also by directly augmenting the genotoxic potential of the oral microbiome (Semiz et al., 2025). Furthermore, poor oral hygiene, characteristic of periodontitis, increases the microbial burden and, consequently, the total acetaldehyde-producing capacity of the oral cavity (Nieminen and Salaspuro, 2018). These observations position microbial acetaldehyde metabolism as a plausible, testable mechanism linking periodontal disease to lung cancer and warrant further investigation through longitudinal microbiome-metabolome studies (**Figure 2**).

**Figure 2:**
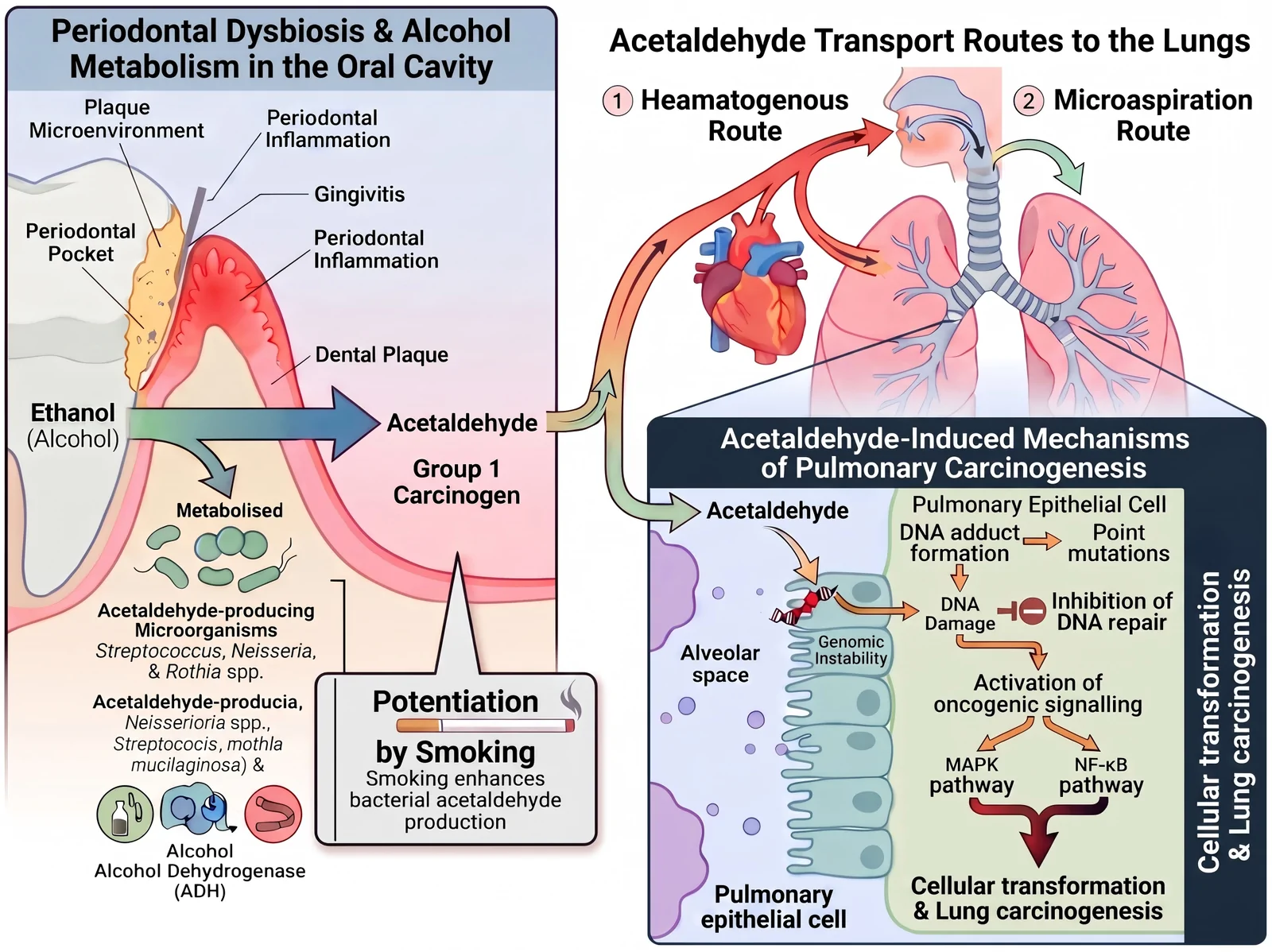
Schematic illustration of microbial acetaldehyde metabolism as a genotoxic mechanism linking periodontal disease to lung cancer. Periodontal dysbiosis promotes the overgrowth of acetaldehyde-producing oral microorganisms (e.g., Neisseria spp., S. mitis, R. mucilaginosa) that possess alcohol dehydrogenase (ADH). These bacteria metabolize ethanol to acetaldehyde, a Group 1 carcinogen. Acetaldehyde induces DNA adduct formation, point mutations, genomic instability, and activates oncogenic MAPK/NF-κB signaling while inhibiting DNA repair. Smoking potentiates this pathway by enhancing bacterial acetaldehyde production. Acetaldehyde reaches the lungs via microaspiration or hematogenous routes, causing direct DNA damage in pulmonary epithelial cells and potentially driving lung carcinogenesis. This mechanism operates independently of, and synergistically with, inflammation-driven pathways.

## 5 Future research directions and prospects

Despite accumulating epidemiological evidence supporting a positive association between periodontal disease and lung cancer, several critical gaps remain to be addressed in future research. Precisely quantifying smoking exposure remains a priority methodological challenge. Current studies predominantly rely on self-reported smoking status or pack-year estimates, which are susceptible to recall bias and fail to capture the full complexity of smoking history, including duration, intensity, and time since cessation. Future cohort studies should incorporate objective biomarkers of tobacco exposure, such as serum cotinine or urinary nicotine metabolites, to better control for confounding. Furthermore, the inconsistent findings among non-smokers underscore the need for large-scale studies with stringent exclusion of former smokers and secondhand smoke exposure, as these factors may still introduce residual confounding even within “non-smoking” populations.

Whether the observed association reflects a causal relationship requires further investigation through advanced causal inference methodologies. Mendelian randomization studies, which leverage genetic variants as instrumental variables for periodontal disease and tooth loss, could provide valuable evidence for or against a causal relationship, with reduced susceptibility to confounding. Additionally, the application of mediation analysis could help disentangle the direct effects of periodontal disease from those mediated through smoking-related pathways or other intermediate factors, such as systemic inflammation.

The biological mechanisms linking periodontal disease to lung cancer warrant further elucidation. While the three mechanistic pathways—microbial translocation, chronic inflammation, and epigenetic modification—have been proposed, their temporal sequence and relative contributions remain poorly defined. Longitudinal multi-omics studies integrating metagenomic, transcriptomic, and epigenomic profiling from the same individuals across multiple time points would enable reconstruction of dynamic molecular trajectories linking periodontitis to pulmonary carcinogenesis. In particular, the role of *Fusobacterium nucleatum* as a direct oncogenic driver requires validation through mechanistic experiments, such as organoid models or gnotobiotic animal systems, to confirm its causal role rather than relying on mere correlational observations.

The translational potential of periodontal interventions for lung cancer prevention should be explored. Although randomized controlled trials evaluating the effect of periodontal treatment on lung cancer incidence would be ethically and practically challenging due to the long latency period of cancer development, surrogate endpoint trials could assess the impact of intensive oral hygiene measures on intermediate biomarkers, such as circulating inflammatory markers, immune cell profiles, or epigenetic signatures. Preliminary intervention studies assessing changes in lung function following improvements in oral hygiene are already underway and may provide proof of concept for the biological plausibility of the oral-lung axis.

Integrating periodontal health assessment into lung cancer risk prediction models represents a promising direction for clinical translation. If the independent contribution of periodontal disease to lung cancer risk is confirmed with greater certainty, incorporating oral health parameters—such as clinical attachment loss, tooth count, or salivary microbiome profiles—into existing risk stratification tools could improve their predictive performance, particularly in never-smokers who currently lack robust risk stratification. In this context, oral fluid biomarkers and chair-side diagnostic tools offer a particularly attractive approach for oncology settings. Unlike comprehensive periodontal examinations, which require specialized training and equipment, matrix metalloproteinase-8 (MMP-8), a key biomarker of active periodontal tissue degradation, can be rapidly measured from saliva or gingival crevicular fluid using point-of-care tests. Elevated MMP-8 levels have been consistently associated with both periodontitis progression and lung cancer risk, suggesting its potential as a cross-disease screening biomarker. Other promising oral biomarkers include IL-1β, IL-6, TNF-α, and antibodies against Porphyromonas gingivalis, all of which reflect the inflammatory burden of periodontitis and may independently contribute to risk stratification. Applying such chair-side diagnostic tools in oncology clinics would require validation in diverse populations and careful consideration of cost-effectiveness, but they offer a pragmatic pathway to incorporate oral health into comprehensive lung cancer risk management without overburdening clinical workflows.

Integrating periodontal health assessment into lung cancer risk prediction models represents a promising direction for clinical translation. If the independent contribution of periodontal disease to lung cancer risk is confirmed with greater certainty, incorporating oral health parameters—such as clinical attachment loss, tooth count, or salivary microbiome profiles—into existing risk stratification tools could improve their predictive performance, particularly in never-smokers who currently lack robust risk stratification. This integration would require validation in diverse populations and consideration of the cost-effectiveness and feasibility of oral health screening in non-dental settings.

## 6 Conclusion

In conclusion, substantial epidemiological evidence supports a significant positive association between periodontal disease and lung cancer risk, with dose-response relationships across clinical severity and tooth loss. The oral-lung axis supports biological plausibility through microbial translocation, chronic inflammation, immune dysregulation, and epigenetic modifications. However, the independent causal nature remains incompletely established due to the strong confounding effect of smoking. While the association persists after adjustment in most studies, inconsistent findings among never-smokers and attenuated effect sizes suggest residual confounding or effect modification. Smoking may synergistically amplify risk. Future research should employ refined exposure measurement, causal inference methods, and multi-omics approaches to further test the causality hypothesis. Although routine periodontal interventions for lung cancer prevention are not yet justified, incorporating oral health into comprehensive risk assessment may offer a complementary strategy. Ultimately, elucidating this relationship will inform both mechanistic understanding and interdisciplinary prevention bridging dentistry and oncology.
